# Accurate quantification of heart valve regurgitation in all four heart valves simultaneously using 3D velocity-encoded MRI with retrospective valve tracking

**DOI:** 10.1186/1532-429X-11-S1-O93

**Published:** 2009-01-28

**Authors:** Jos JM Westenberg, Stijntje D Roes, Rob J van der Geest, Sebastiaan Hammer, Nina Ajmone Marsan, Jeroen J Bax, Albert de Roos, Johan HC Reiber

**Affiliations:** Leiden University Medical Center, Leiden, Netherlands

**Keywords:** Aortic Valve, Mitral Valve, Heart Valve, Right Ventricle, Tricuspid Valve

## Introduction

In regurgitant heart valves, surgical decision-making is based on the severity of the regurgitation through the particular valve. Conventional two-dimensional (2D) one-directional velocity-encoded (VE) MRI is routinely used for flow assessment, but this technique has been shown to be inaccurate and correlation between the net flow volumes through the valves is weak, even in the absence of regurgitation. 2D one-directional VE MRI is limited because the position and angulation of the acquisition plane cannot be adapted to the valve motion and the direction of the inflow and regurgitant jet.

## Purpose

Three-dimensional (3D) 3-directional VE MRI with retrospective valve tracking during offline analysis is introduced for flow assessment through all heart valves simultaneously. This technique is validated in phantoms and applied in 14 volunteers without and 23 patients with valve regurgitation.

## Methods

**Figure 1:**
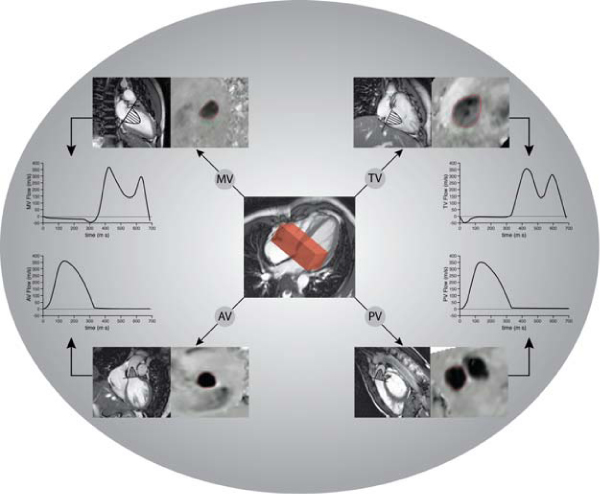
**3D 3-directional velocity-encoded MRI is performed at the basal level of the heart**. In offline analysis, retrospective valve tracking for each of the heart valves can be performed, resulting in flow at the mitral valve (MV), tricuspid valve (TV), aortic valve (AV) and pulmonary valve (PV).

MRI was performed on a 1.5 T Gyroscan ACS/NT15 MRI (Philips, Best, the Netherlands). A 3D 3-directional VE MRI sequence was designed (3D volume scan with slab thickness 48 mm, acquisition voxel size 2.9 × 3.8 × 4.0 mm^3^, three-directional velocity sensitivity 150 cm/s, with 30 phases reconstructed during one average cardiac cycle, Echo Planar Imaging factor 5, with free-breathing in vivo) and tested in stationary flow phantoms and in a phantom simulating harmonic left ventricular filling. From the 3D velocity-data, through-plane velocity was reformatted offline for each valve plane using two orthogonal reformat-guides per plane (i.e., for mitral valve (MV): 2- and 4-chamber of the left ventricle (LV); tricuspid valve (TV): 2- and 4-chamber of the right ventricle (RV); aortic valve (AV): two orthogonal views of LV outflow tract; pulmonary valve (PV): two orthogonal views of the RV outflow tract) (Figure 1).

In 14 volunteers without regurgitation and in 23 patients with single or multiple valve regurgitation proven on echocardiography, trans-valvular flow was assessed at all four valves using 3D 3-directional VE MRI. Regurgitation was quantified. Correlation between the net flow volumes per valve was examined and differences were studied.

## Results

**Figure 2:**
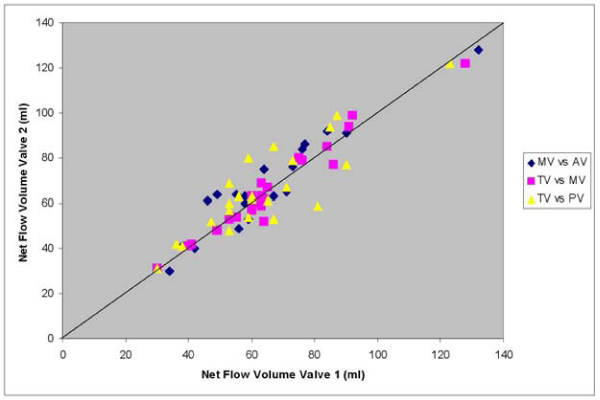
**Net flow volumes for mitral valve (MV) vs. aortic valve (AV), tricuspid valve (TV) vs. MV and TV vs. pulmonary valve (PV) in 23 patients with valve regurgitation**.

Validation in phantoms showed less than 5% error in flow. In vivo, mean scan time = 4.2 ± 0.8 min at a mean heart rate of 67 ± 12 beats per minute. In volunteers, comparison of the net flow volumes through the four valves showed strong correlation with a only small differences between AV and MV and between PV and TV (statistically significant but clinically non-significant) and with small confidence intervals (Table 1). In patients, also strong correlation between the net flow volumes per valve were found (Figure 2), with no significant biases. Mean regurgitant fraction for MV = 12 ± 8% (range: 4–29%), TV = 10 ± 7% (range: 2–25%), AV = 2 ± 2% (range: 0–5%) and PV = 3 ± 3% (range: 0–10%).

**Table 1:** Statistical results

		MV-AV	MV-TV	TV-PV
Volunteers (n = 14)	Pearson correlation	0.96	0.97	0.96
	p-value t-test	0.01	0.31	0.01
	Mean difference	6 ± 7 ml	-2 ± 5 ml	6 ± 6 ml
	Confidence interval	-7 – 19 ml	-12 – 9 ml	-6 – 17 ml
Patients (n = 23)	Pearson correlation	0.95	0.98	0.88
	p-value t-test	0.07	0.75	0.30
	Mean difference	3 ± 6 ml	0.3 ± 4 ml	2 ± 10 ml
	Confidence interval	-10 – 16 ml	-8 – 9 ml	-17 – 22 ml

## Conclusion

3D VE MRI provides the true trans-valvular flow for all four heart valves from a single acquisition in less than 5 minutes scan time. Regurgitation can be quantified accurately, providing essential information for surgical decision-making.

